# Multipurpose deep learning-powered UAV for forest fire prevention and emergency response

**DOI:** 10.1016/j.ohx.2023.e00479

**Published:** 2023-09-20

**Authors:** Tejas Rathod, Vinay Patil, R. Harikrishnan, Priti Shahane

**Affiliations:** Symbiosis Institute of Technology, Pune Campus, Symbiosis International Deemed University, Pune, India

**Keywords:** UAV, Deep learning, GANs, Forest fire prediction

## Abstract

This paper presents a customized UAV designed for rescue and safety purposes in the forest sector. The UAV features a durable F450 frame quadcopter with four 1000KV brushless motors and a KK2.1 Flight Control Board for stability and manoeuvrability with a runtime of 90 min. It incorporates a Raspberry Pi camera for real-time video streaming, enabling efficient identification of individuals in need of assistance. The GSM module allows contactless communication, ensuring streamlined and safe interaction. A motor controls the lid of the customizable first aid kit box, facilitating efficient aid delivery. The Neo-6 M GPS module provides accurate localization of the drone and individuals in distress with a horizontal position accuracy of 2.5 m. The UAV collects temperature and humidity data using the DHT 11 sensor having +/- 2 degreesC and +- 5% accuracy respectively. This sensor employs advanced deep learning models, including artificial neural networks (ANN) and generative adversarial networks (GANs), for real-time forest fire prediction with an accuracy of 90.7 % The integration of GANs enhances accuracy through synthetic data generation. Moreover, all these components are interfaced using a Raspberry Pi4 and a GUI, providing a smooth user control experience and end-to-end information for quick and effective emergency response.

## Graphical abstract

**Figure:**
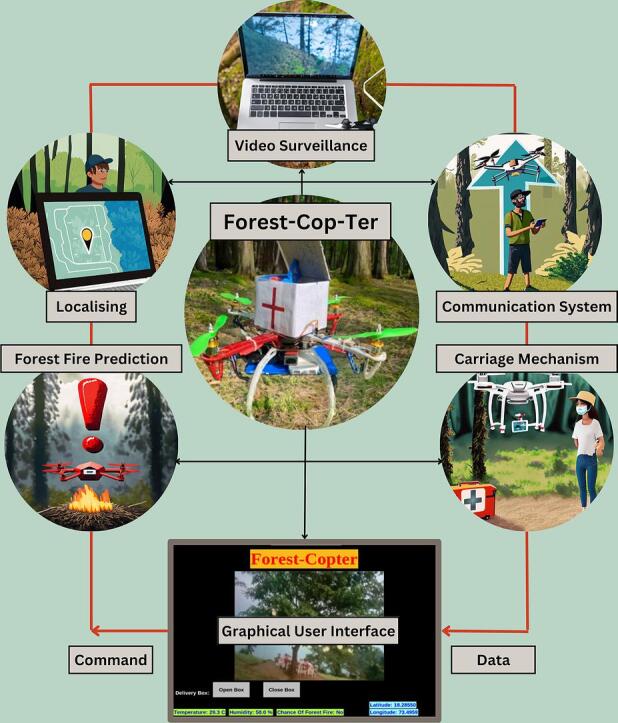

## Nomenclature

**Acronyms**
*UAV*: *Unmanned Aerial Vehicle*
*GSM*: *Global System for Mobile Communications*
*GPS*: *Global Positioning System*
*ANN*: *Artificial Neural Network*
*GANs*: *Generative Adversarial Networks*
*GUI*: *Graphical User Interface*
*DJI*: *Da-Jiang Innovations*
*APN*: *Access Point Name*
*BPN*: *Back Propagation Network*
*GND*: *Ground*
*AI*: *Artificial Intelligence*
*KV*: *Kilo Volt*
*BAT*: *Battery*
*PI*: *Proportional-Integral*
*RBFN*: *Radial Basis Function Network*
*DLVQ*: *Dynamic Learning Vector Quantization*
*TWR*: *Thrust to Weight Ratio*
*CG*: *Centre of Gravity*
*ESC*: *Electronic Speed Controller*
*AT*: *Attention*
*UART*: *Universal Asynchronous Receiver/Transmitter*
*GPIO*: *General-Purpose Input/Output*
*PWM*: *Pulse Width Modulation*
*DHT*: *Digital Humidity and Temperature*
*PLA*: *Polylactic Acid*
*CH*: *Channel*
*ACC*: *Accelerometer*
*PIL*: *Python Imaging Library*

## Specifications table

**Table:** 

**Hardware name**	*Forest-Cop-Ter*
**Subject area**	• *Engineering and material science* • *Environmental, planetary, and agricultural sciences*
**Hardware type**	• *Electrical engineering and computer science* • *Mechanical engineering and materials science* • *Artificial Intelligence*
**Closest commercial analog**	*No commercial analog is available.*
**Open-source license**	*CC BY 4.0*
**Cost of hardware**	*$ 315*
**Source file repository**	https://doi.org/10.17632/46x948gzkt.1
**OSHWA certification UID**	*IN000044*

## Hardware in context

There are a lot of concerning issues growing day by day in the forest sector. The first one is a major threat to both environment and humans which is a forest fire. As stated by a survey, fires are burning faster and hotter than ever before unlike what used to take place in the 90 s. In 2022, thousands of people had to evacuate in Northern Algeria where almost 140 people suffered death due to the extreme fire. A more alarming report suggested that an event like Australia's wildfire could occur by the end of this century [1]. In 2022, the US forest sector reported at least 7,534,403 acres burned due to forest fires [2]. On March 2022 India reported 340 incidences of forest fire [3]. Forests are quite dense and hard to keep track of the path we come in and has increased chance of getting lost. An incident was reported of a hiker that went missing on an attempt to hike Mount Redington in July 2013. The search and rescue operation was termed to be one of the biggest in the state's history as it took place over two years later only to be found by a forest surveyor collapsed in a tent [4]. Occupational work taking place in the forest is deemed one of the most dangerous ones [5]. The incidence rate of forestry is one of the highest [6]. Especially in the case of calamities which have increased in recent years, an increased risk of accidents for forest workers is to be expected [7], and this accident rate is expected to be doubled [8]. This tells us how often people are prone to get injured in the forest and need of quick medical assistance and effective emergency response.

Thus, to tackle these problems at once a multipurpose UAV is designed. This UAV is an F450 quadcopter with a KK 2.1 Flight Controller Board as its brain to receive and transmit signals using a Fly Sky 6CH controller. In terms of popular flight controller board options Ardupilot [9] and PX4 platforms [10] are one of the popular options to choose from as they wide range of customization options and improved accuracy when compared to the KK 2.1 Flight controller board but come with a higher cost. Proprietary options to the F450 Quadcopter include DJI Matrice 600 Pro [11] and the Autel Robotics EVO II [12], both have advanced features with excellent flight accuracy. However, these options may come at a higher cost and may have limitations in terms of customization. This drone is customized to be capable of five applications all interfaced with Raspberry Pi 4. First is the surveillance system to keep a watch in the forest by using a Raspberry Pi Camera to get high-definition video surveillance. In competition with this, popular choices are the Arduino camera module [13] and the BeagleBone Black camera cape [14]. These devices are known to use a processor board to control the camera and provide real-time surveillance. In terms of actual products DJI drones are quite famous for aerial photography and specially designed cameras for drones - GoPro cameras [15], [26] also can transmit and stream live video. Ultimately, the decision came down to going for a camera that is cost-effective and gives a decent enough performance for real-time video transmission which the Raspberry Pi camera is capable of. Second is the communication system built to assist individuals who may have lost their way or need guidance on a specific problem. This system is made contactless and achieved using a GSM module. The reason for this is to prevent any harm to the drone and prevent the case of a person being injured and not able to use the keypad. A similar open-source communication system would be the LoRa [16] module which also serves the purpose of long-range communication, but GSM technology edges this technology in terms of providing a much more larger coverage area. However, commercial drones do have advanced communication systems built which outperform the GSM technology. The third is the GPS location of the UAV to keep track of the drone and to also send a rescue team to a particular location when encountering a particular situation in need of immediate expert assistance. This task is achieved using the Neo-6 M GPS module as it is a popular and cost-effective option with a low power consumption advantage. Other similar open hardware options for GPS modules include U-Blox NEO-M8N [17] and Adafruit Ultimate GPS Breakout [18], but of all DJI's NAZA GPS module [19] is well known for both accuracy and reliability. Fourth is the carriage mechanism for the delivery of payloads. This mechanism is dedicated solely to the purpose of providing medical assistance as a first aid toolkit box. This toolbox's gears are 3D printed and for functionality rely on a servo motor instead of a stepper motor as it offers greater precision. In comparison to this gimbal motors [20] also offer similar accuracy but come at a higher cost. Lastly, there is a temperature-humidity sensor, DHT11 interfaced to collect real-time data that is used to predict forest fires in real-time using a deep learning model which is described in detail in the later part of the paper. This model was trained on both real-time and synthetic data to improve upon the performance and tackle the problem of scarcity of publicly available datasets of forest fires. This synthetic data was generated using GANs. Other proprietary hardware options include the BME280 temperature-humidity sensor [21] or the Si7021 temperature-humidity sensor [22], [27]. Both of these hardware outperform the DHT 11 sensor in terms of accuracy and range of measuring but do come at a higher cost. Moreover, all these features are made accessible through a simple GUI which provides a smooth experience for the user in operating the drone.

## Hardware description

This UAV is a highly customized piece of hardware designed for use in the forest sector for rescue and safety purposes. It is designed to carry out multiple tasks at a single time, making it stand out in terms of novelty and ease of use.

### Quadcopter

A F450 frame is chosen for the quadcopter because of its durability and stability. Four 1000KV brushless motors are connected to Brushless motor speed controller with KK2.1 Flight Control Board as its brain. Together, these parts lift, regulate movement, and enable the quadcopter to be highly manoeuvrable and sensitive to input. With the help of those strong brushless motors and the light-weight frame used, high TWR of 2:1 is achieved. As a result, the quadcopter can carry a sizable payload without compromising its stability. The quadcopter's CG is precisely calculated to ensure stable flight in any orientation. The quadcopter also has a FlySky 6CH Controller, which relays instructions to the flight control board. This offers a high degree of control and accuracy, enabling the quadcopter to carry out a variety of tasks. The KK2.1 Flight Control Board has a significant advantage over other flight control boards in terms of usability. Anyone, not just experts, can fly the quadcopter thanks to the built-in software and LCD screen, which offer easy setup and installation. Additionally, the quadcopter can handle a variety of tasks and payloads thanks to its high TWR and precise CG.

### Surveillance system

The UAV is equipped with a Raspberry Pi camera to locate a person in need of assistance. This camera was picked for its affordability, ease of interfacing it with Raspberry Pi 4 and ability to stream live high-quality videos in real time. Thus, serving the purpose of identifying a purpose even in a dense forest. This approach outperforms the pre-existing methods such as aerial surveillance using manned aircrafts or sending in ground-based search teams in terms of efficiency, time-saving, quick response, cost-saving and minimal risk of human life. With these features this application can be easily extended to monitoring of wildlife or aerial photography.

### Communication system

The drone is equipped with a GSM module which gives a unique feature that allows for contactless communication with a person in need of assistance. The GSM module is connected to the Raspberry Pi's UART and utilizes the pySerial library along with AT commands to provide guidance and assistance. Unlike traditional GSM modules that require a keypad for dialling or picking up a call, the GSM module allows for a more streamlined and safe process, as it eliminates the need for a person to interact with the keypad, especially in cases where the person may be injured and unable to use the keypad properly. This feature sets this drone apart from pre-existing methods that rely on keypad-based communication, and offers a distinct advantage in terms of ease of use and safety. Additionally, it can be used to further develop designs in areas where keypad-free communication is essential such as emergency rescue scenarios.

### Carriage mechanism

The drone is equipped with a customizable first aid kit box that can be used to aid a person in need of assistance. This feature is made possible by the utilization of a servo motor that is connected to the Raspberry Pi. Using PWM and the RPi.GPIO library in Python programming, the servo motor is able to accurately position the lid of the first aid box, making it easy to access the contents inside. This is a unique feature as compared to traditional first aid kits that are manually opened, as it allows for more precise and efficient delivery of aid. Additionally, the use of a servo motor also allows for more cost-effective solution compared to using other types of motors. The delivery mechanism includes the 3D-printed gears that help open and close the delivery box mounted on the drone. One of the gears is fixed to the lid of the box and the other is connected to the shaft of the servo motor. These gears are designed on the Fusion 360 by Autodesk and 3D printed. These gears are meshed using the teeth present on both gears which can lead to rotary motion. These gears help in the smooth operation of the box which is controlled by the drone user through the widgets or buttons present on the GUI to open and close.

### Localising

The Neo-6 M GPS module is used to retrieve the location of the drone to keep its track which goes hand in hand of obtaining the location of the person in need of assistance and sending this to the emergency-response team for extraction. This module is interfaced with the Raspberry Pi4 which gives us the capability in gaining precise location throughout the usage of the drone via the GUI designed. When compared with popular alternatives like U-Blox GPS module it edges them over two parameters that is affordability and higher precision in real time. Moreover, using this feature it’s application can be also extended in locating endangered species or mapping an area.

### Forest fire prediction

This drone utilizes state of the art deep learning model built on latest techniques like ANN and GANs to predict forest fires using just two parameters. What separates us from the rest is the use of GANs to generate synthetic data to train the model to improve its real-time accuracy and tackle the constraint of limited dataset availability. DHT 11 sensor is interfaced with Raspberry Pi 4 to collect real time data which is processed though the model and predictions are made in real time. Although not best in terms of range and accuracy in collecting data the sensor does give performance apt enough in terms of its affordability. Overall, this forest fire prediction system is a cost effective, precise enough system for real time usage competing with other available models based on just two parameters.

This UAV serves as a multipurpose drone in the forest sector for rescue and safety purposes. Its ease of use and ability to carry out multiple tasks at a single time is what makes it stand in terms of novelty. The drone is designed such that it carries out the whole rescue operation in terms of aiding the forest department to help a person stranded in the forest. The camera helps find a person, once the person is found the GSM helps the drone operator converse with the person to provide assistance, and the attached first aid kit box can be used to aid the person’s injury if any, using the GPS the person’s location is obtained to send in help or rescue that person from its location; all these applications accessed and controlled over a simple GUI. The drone not only aids in terms of helping a human but also serves in aiding the forest while performing surveillance. Many AI models use multiple parameters to predict a forest fire but aligning with the design of this drone that is cost-effective only two parameters were used aiming to better with the given circumstances. This paper aims to contribute in terms of bettering the standards of the forest department with the help of a cost-effective UAV and carrying out their usual tasks quickly and efficiently with the help of new-age technology. Although this drone is customized to fulfil the needs of the forest department, this does not limit its ability to aid in other sectors which could come useful for other researchers as well:

1. The drone’s surveillance can be enhanced by AI with state-of-the-art image processing techniques to classify for example a species, area for mapping, finding defects in large infrastructures and much more.
2. Using it’s rescue operation ability this drone can aid the fire-department to assist the civilians safely out of a danger zone.
3. Researchers can also integrate ROS in it for survey purposes of a particular area.
4. This drone can be used to monitor the weather also be it for say agricultural purposes to enhance the yield.
5. Using GANs researchers can improve upon areas with limited availability of data.

## Design files summary

**Table:** 

**Design filename**	**File type**	**Open-source License**	**Location of the file**
*GSM_testing.py*	*micropython file*	*CC BY 4.0*	https://doi.org/10.17632/46x948gzkt.1
*camera_testing.py*	*micropython file*	*CC BY 4.0*	https://doi.org/10.17632/46x948gzkt.1
*gps_testing.py*	*micropython file*	*CC BY 4.0*	https://doi.org/10.17632/46x948gzkt.1
*servo_testing.py*	*micropython file*	*CC BY 4.0*	https://doi.org/10.17632/46x948gzkt.1
*temperature_sensor_testing.py*	*micropython file*	*CC BY 4.0*	https://doi.org/10.17632/46x948gzkt.1
*forest-copter.py*	*micropython file*	*CC BY 4.0*	https://doi.org/10.17632/46x948gzkt.1
*rpi_camera_surveillance_system.py*	*micropython file*	*CC BY 4.0*	https://doi.org/10.17632/46x948gzkt.1
*Servo_Holder.stl*	*3D (STL)*	*CC BY 4.0*	https://doi.org/10.17632/46x948gzkt.1
*gear_mechanism.step*	*CAD File*	*CC BY 4.0*	https://doi.org/10.17632/46x948gzkt.1
*gear_mechanism_mount.step*	*CAD File*	*CC BY 4.0*	https://doi.org/10.17632/46x948gzkt.1
*FF_Prediction.ipynb*	*IPython Notebook File*	*CC BY 4.0*	https://doi.org/10.17632/46x948gzkt.1
*Final_model.h5*	*Model File*	*CC BY 4.0*	https://doi.org/10.17632/46x948gzkt.1
*SyntheticData.ipynb*	*IPython Notebook File*	*CC BY 4.0*	https://doi.org/10.17632/46x948gzkt.1
*forestfire_prediction.py*	*micropython file*	*CC BY 4.0*	https://doi.org/10.17632/46x948gzkt.1
*forestfires.csv*	*Data File*	*CC BY 4.0*	https://doi.org/10.17632/46x948gzkt.1
*forestfire_augmented.csv*	*Data File*	*CC BY 4.0*	https://doi.org/10.17632/46x948gzkt.1
*rnet.txt*	*text file*	*CC BY 4.0*	https://doi.org/10.17632/46x948gzkt.1

Below is a brief description of the file listened above:

• GSM_testing.py: This micropython file contains the code to test the Global System For Mobile communication (GSM) Module. This file can make a call to a specified number in code.
• camera_testing.py: This micropython file contains the code to test the camera module. It will show the camera feed.
• gps_testing.py: This micropython file contains the code to test the Global Positioning System(GPS) Module. It will give the latitude and longitude as output.
• servo_testing.py: This micropython file contains the code to test the servo motor installed. It will rotate its shaft by 180 degrees and go back to 0 degree.
• Temperature_sensor_testing.py: This micropython file contains the code to test the temperature sensor. It will give the temperature and humidity level of the surroundings.
• forest-copter.py: This micropython file contains the code to the Graphical user Interface (GUI). After running this file all sensors and modules will start working.
• rpi_camera_surveillance_system.py: This micropython file contains the code to the Graphical user Interface (GUI).
• Servo_Holder.stL: This 3D STL file contains the 3D model which is 3D printed to hold the servo motor.
• gear_mechanism.step: This CAD file contains the 3D model which is 3D printed and used to open and close the delivery box.
• gear_mechanism_mount.step: This CAD file contains the 3D model which is 3D printed and mounted on a servo motor shaft.
• FF_Prediction.ipynb: This Ipython Notebook File preprocesses the data then trains and tests the model.
• Final_model.h5: This is the final model file for predicting the chances of forest fire.
• SyntheticData.ipynb: This Ipython Notebook File which generates synthetic data for model training and testing, it also compares the synthetic data with the original data and gives a detailed comparative analysis for both the datasets.
• forestfire_prediction.py: This micropython file contains the code for prediction of chances of forest fire
• forestfires.csv: This file contains the meteorological data of forest fires which we got from the UCI Machine Learning repository[].
• forestfire_augmented.csv: This file contains the synthetic data which was generated using SyntheticData.ipynb. This dataset was generated using the concept of Generative Adversarial networks(GANs).
• rnet.txt: This txt file is used for the configuration of Peer Software which will be used to access the internet using GSM module.

## Bills of materials summary

**Table:** 

**Designator**	**Component**	**Number**	**Cost per unit - USD**	**Total cost - USD**	**Source of materials**	**Material type**
*P01*	*RaspberryPi4*	*1*	*70*	*70*	https://robu.in/	*Semiconductor*
*P02*	*Orange 5200mAh Battery*	*1*	*42*	*42*	https://robu.in/	*Non-specific*
*P03*	*Neo6m GPS Module*	*1*	*3.71*	*3.71*	https://robu.in/	*Semiconductor*
*P04*	*RaspberryPi Camera*	*1*	*25.44*	*25.44*	https://robu.in/	*Semiconductor*
*P05*	*GSM Sim900A*	*1*	*13.32*	*13.32*	https://www.amazon.in/	*Semiconductor*
*P06*	*TowerPro Micro Servo SG90*	*1*	*1.93*	*1.93*	https://robu.in/	*Polymer*
*P07*	*DHT11 Temperature Sensor*	*1*	*1.20*	*1.20*	https://robu.in/	*Semiconductor*
*P08*	*Microphone*	*1*	*0.59*	*0.59*	https://robu.in/	*Semiconductor*
*P09*	*Speaker*	*1*	*0.55*	*0.55*	https://www.electronicscomp.com/	*Semiconductor*
*P10*	*DJI F450 Drone Frame*	*1*	*20.84*	*20.84*	https://www.amazon.in/	*Polymer*
*P11*	*Simonk 30A ESC*	*4*	*4.85*	*19.39*	https://robu.in/	*Semiconductor*
*P12*	*1000KV Brushless Motor*	*4*	*3.64*	*14.54*	https://robu.in/	*Metal*
*P13*	*8x4.5″ Propeller*	*4*	*1.20*	*4.80*	https://robu.in/	*Polymer*
*P14*	*KK2.1 Flight Controller*	*1*	*50.90*	*50.90*	https://robu.in/	*Semiconductor*
*P15*	*Flysky 6CH Controller*	*1*	*30.28*	*30.28*	https://robu.in/	*Semiconductor*
*P16*	*PLA*	*1*	*800*	*800*	https://robu.in/	*Polymer*
*P17*	*USB to Type C cable*	*1*	*9.69*	*9.69*	https://robu.in/	*Non-specific*
*P18*	*Dual USB Output*	*1*	*2.42*	*2.42*	https://robu.in/	*Semiconductor*
*P19*	*Pole Wire Connector Terminal Block*	*1*	*1.56*	*1.56*	https://robu.in/	*Semiconductor*
*P20*	*Jumper Wires*	*1*	*1.09*	*1.09*	https://robu.in/	*Semiconductor*
*P21*	*Small BreadBoard*	*1*	*0.35*	*0.35*	https://robu.in/	*Semiconductor*
*P22*	*Cardboard*	*1*	*0.61*	*0.61*	*Local store*	*Composite*
*P23*	*Sheet Metal*	*1*	*1.21*	*1.21*	*Local store*	*Metal*
*P24*	*Spacers*	*4*	*0.12*	*0.48*	*Local store*	*Polymer*

### Build instructions

**Fig. 1:**
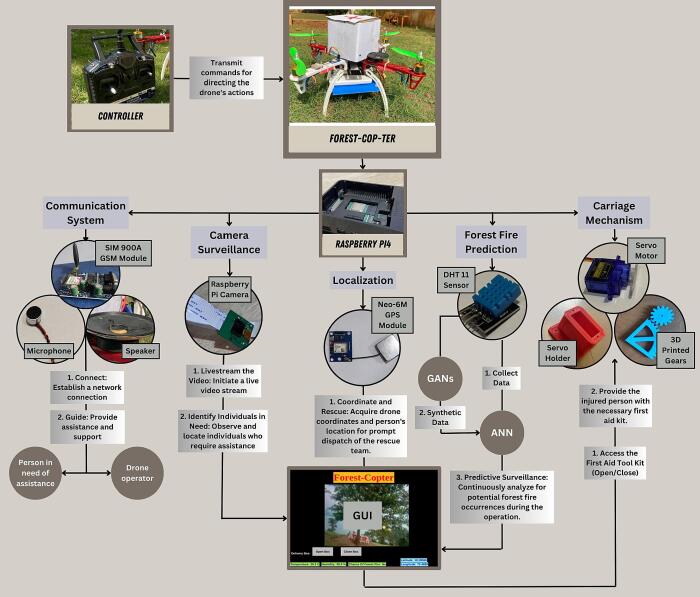
Operational Framework of the Quadcopter's Architecture.

*In the following section, we present a concise and structured guide outlining the step-by-step build instructions for recreating the designed quadcopter. This walkthrough provides a comprehensive approach to assembling and configuring the components, ensuring a successful replication of the quadcopter system. Additionally, below* Fig. 1 *illustrates an overview of the quadcopter's operational framework, showcasing its key components and highlighting its versatile applications.*

### Drone Assembly

The step-by-step procedure to assemble the drone is to start with the mounting of 1000KV Brushless motors(P12) to the F450 quadcopter frame(P10), using the M3 screws provided with the motor.

**Fig. 2:**
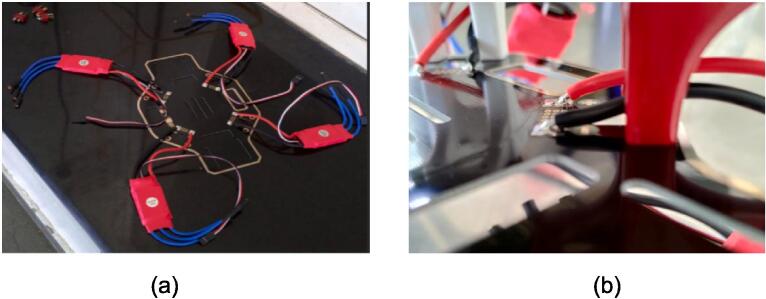
Assembling of Drone (a) Soldering all the ESCs and power wire with the bottom pad, (b) soldering of red wire to positive pad and black wire to the negative pad. (For interpretation of the references to colour in this figure legend, the reader is referred to the web version of this article.)

*Solder the ESC(P11) and power wire to the power pads (bottom board) of the F450 Frame as shown in* Fig. 2 *(a). Solder red wire of ESC to the positive pad denoted by the + sign on the board and black wire of ESC to the negative pad denoted by the - sign on the board as shown in* Fig. 2 *(b) Make sure this soldering does not cause any kind of short circuit. Insert the power wire inside the output of Pole Wire Terminal block(P19). It is a versatile component used for secure and efficient electrical connections. With multiple connection points, it simplifies installation, enhances flexibility, and ensures safety. Make sure that blue stands for positive or negative and orange stands for the opposite terminal of blue. If blue stands for positive than orange stands for negative. Make proper connections to avoid any damage to the components.*

**Fig. 3:**
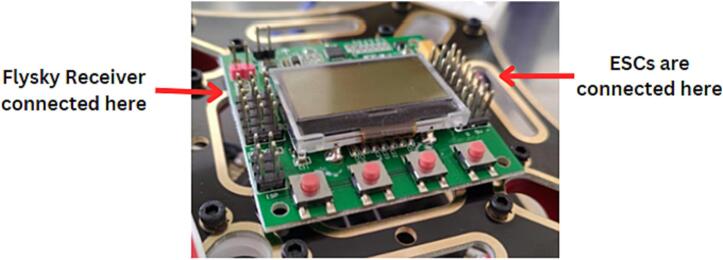
Mounting of flight controller on the top board.

Mount the KK2.1 flight controller(P14) on the top of the F450 quadcopter frame (top board) as shown in Fig. 3.

**Fig. 4:**
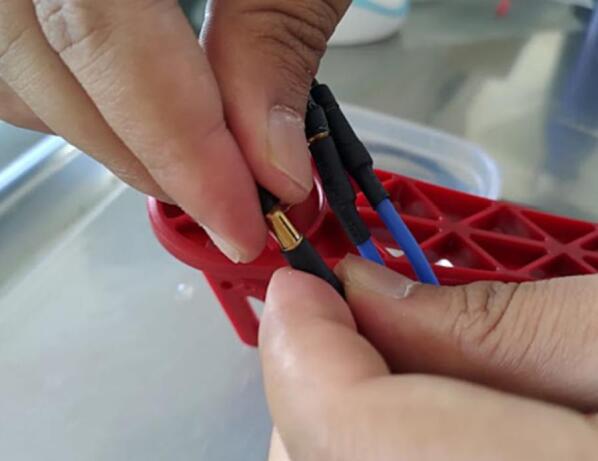
Connecting ESCs to the Brushless Motor.

*Connect ESCs to the motors and connect the signal cable from ESCs to the flight controller, where white wire from ESC is the signal cable, red is the power cable and black is the ground. The arrow marked on the top side of the flight controller gives the front direction of the drone, according to which connection of ESCs is made with the flight controller. Connect the ESCs wires to the right side of flight controller and wires from the Flysky receiver(P15) to the left side of the flight controller as shown in the* Fig. 2 *. Set the quadcopter to fly in the X configuration. So, the top left motor is our Motor 1, top right motor is Motor 2, bottom right motor is Motor 3 and bottom left is Motor 4. Now connect these motor ESCs according to the M1, M2, M3 and M4 pins on the flight controller. Connect the ESCs to the motor, wires will be connected serially leftmost wire is connected to leftmost wire of the motor and similarly for the remaining two wire, refer* Fig. 4 *for the connection.*

**Fig. 5:**
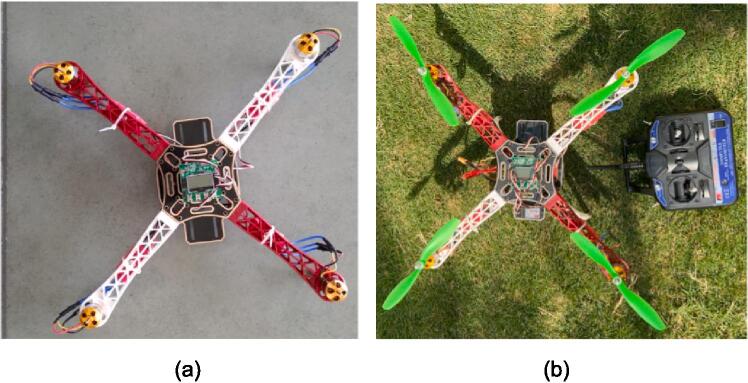
Drone assembly (a) Before calibration of ESCs and flight controller, (b) mounting of propellers after calibrating the ESCs and flight controller.

*The motor will rotate either in clockwise or counter clockwise direction, so by interchanging the last two wires, the polarity can be changed. Connect the Flysky receiver to the flight controller. Connect the signal pin of receiver to the signal pin of the flight controller, +5V pin of receiver to the + 5 V of the flight controller and Ground pin of the receiver to the ground pin of the flight controller. Connect the Flysky receiver such that Channel 1(CH1 on the receiver) of the receiver is connected to the topmost pin on the left side of the flight controller. Similarly Channel 2(CH2 on the receiver) of the receiver is connected to the second pin from the top of the flight controller on the left side as shown in* Fig. 2 *. Similarly, connect the Channel 3, 4, and 5. Now assemble both the boards (top and bottom) to the arms and legs of the quadcopter frame as shown in the* Fig. 5 *(a). After doing the Flight controller calibration and ESC calibration, explained step by step in the operation instructions section. After calibration mount the propellers(P13) on the motors as shown in the* Fig. 5 *(b) and then the drone is ready to fly.*

**Fig. 6:**
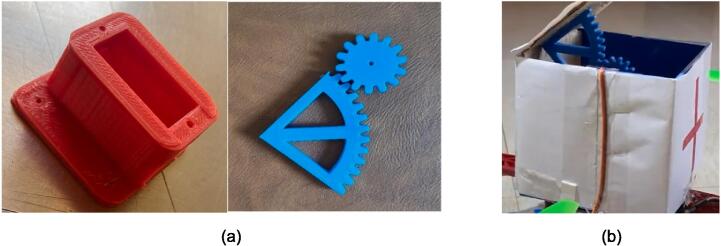
Carriage mechanism (a) 3D printed gears (b) Mounting of these gears on the carriage mechanism.

*As the drone is assembled, mount all the components such as Raspberry Pi, sensors, and carriage mechanism. Attach all the sensor on the arms of the drone. Find a box or with the help of cardboard(P22) make a box of 12x12x12cm dimension. Cut a sheet metal(P23) of 12x12cm dimension and fix it with the base of the box and with the help of spacers(P24) attach the box to the top plate of the drone. With the help of a 3D printer 3D print all the three files containing one spur gear, servo holder and a gear as shown in the* Fig. 6 *(a). Connect the spur gear to the shaft of the motor and insert the servo motor in the holder and stick the other gear such that it is attached to the lid of the box shown in the* Fig. 6 *(b).*

### Raspberry Pi

*First connect the Pole Wire Terminal block output to the input of the Dual USB Output module(P18). Using the USB to type C cable(P17) connect it to the USB port of the Dual USB Output module to power the RaspberryPi4(P01).*

*Connect the Raspberry Pi camera(P04) to the camera module port of the RaspberryPi4. The Neo6M GPS module(P03) consists of Vcc, Gnd, Rx and Tx pins. Connect Vcc pin of GPS module to 5 V of Raspberry Pi4, Ground pin of the GPS module to Ground of Raspberry Pi4, Rx pin of GPS module is not connected, and Tx pins of GPS module is connected to the Rx pin (GPIO 27) of the RaspberryPi4.*

*The DHT11 Temperature sensor(P07) consists of Vcc, Gnd, and data pins. Connect Vcc pin of Temperature sensor to 3.3 V of Raspberry Pi4, Ground pin of the Temperature sensor to the ground of Raspberry Pi4, and data pin of temperature sensor to the GPIO 4 of the RaspberryPi4.*

*The TowerPro Micro Servo SG90 Motor(P06) consists of Vcc, Gnd, and signal pins. Connect Vcc pin of servo motor to 5 V of Raspberry Pi4, Ground pin of the servo motor to the ground of Raspberry Pi4, and signal pin of servo motor to the GPIO 18 of the RaspberryPi4.*

*The GSMSim900A module(P05) consists of Vcc, Gnd, Rx and Tx pins. Connect Vcc pin of GSM module to 5 V of Raspberry Pi4 with the help of breadboard(P21), Ground pin of the GSM module to Ground of Raspberry Pi4, Rx pin of GSM module to the Tx pin (GPIO 14) of the RaspberryPi4 and Tx pins of GSM module to the Rx pin(GPIO 15) of the RaspberryPi4. Now connect the speaker(P09) and microphone(P08) to the ports on the GSM module(P05). There are ports named mic_p and mic_n for Microphone, similarly, connect the positive wire and negative wire of the speaker to spk_n and spk_p respectively.*

**Fig. 7:**
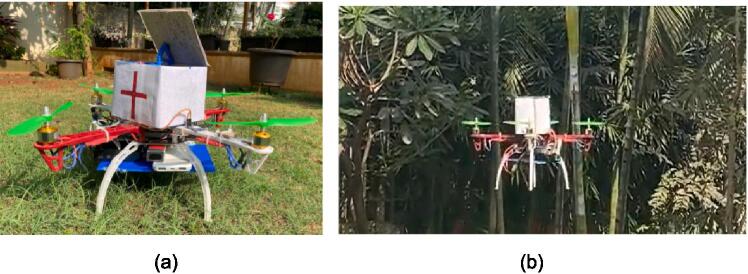
Testing of drone (a) Assembled Drone Model (b) Flying of Assembled drone model.

Below is the image(Fig. 7(a) and (b)) of the drone after attaching all the components, box and 3D printed.

Generate networks:

1. Load on your Google Drive the notebook files: SyntheticData.ipynb, forestfire_prediction.py.
2. Load on your Google Drive forestfires.csv dataset to be used with the SyntheticData.ipynb file in order to generate the synthetic data.
3. Open the file SyntheticData.ipynb with Google Colaboratory, choose runtime session from the Menu and execute.
4. Load on your Google Drive the file generated from step 3 or you can load the forestfire_augmented.csv dataset to be used with forestfire_prediction.py to train the ANN model and make predictions.
5. Repeat step 3 with forestfire_prediction.py, the output model files will be saved in the same directory of the notebook file.

## Operation instructions

### Drone

#### Flysky transmitter and receiver binding

*If the Flysky receiver led is blinking this means that our ESC is not bound with the transmitter. For binding, remove all the connections from the Flysky receiver and connect one of the ESC to the receiver. If the receiver is not bound, then connect the battery(P02) to the power cable of the frame soldered earlier and power the ESCs. In the Flysky receiver the outer pins are the ground pins, middle pins are the Vcc pins and inner pins (pins towards the antenna) are the signal pins.*

*Now connect one of the ESC cables which will go to the flight controller to Channel 1(written as CH1 on the receiver) of the receiver and short the signal and ground pin of the receiver at the BAT channel. If the receiver is not bound, then the led on the receiver will blink continuously. For binding make the trim levels of the transmitter to middle and press the bind button on the transmitter and turn on the transmitter by keeping the bind button pressed. After binding the receiver LED on the receiver will not blink which means that the transmitter and receiver are bound to each other.*

## Now disconnect the power and turn off the Flysky transmitter

### Flight control calibration

*There are four buttons present on the flight controller, first button(denoted as S1 on the flight controller) from left is used to go back in the menu, second button(denoted as S2 on the flight controller) from left is used to go up, third button(denoted as S3 on the flight controller) from left is used to go down, and fourth button(denoted as S4 on the flight controller) from left is used to go inside the menu. First, turn on the Flysky transmitter, and then power the quadcopter by connecting the battery to the power cable soldered.*

*Press the S4 button and navigate to load motor layout using S2 and S3 buttons and select the Quadcopter X mode using the S4 button then it will show the configuration of our drone such that the motor 1 and motor 3 should rotate clockwise and motor 2 and 4 should rotate counter clockwise. Before flying make sure the motors are rotating properly according to the X configuration.*

*Now calibrate the Accelerometer (ACC), for this make sure the drone is on the flat surface and press calibrate on the flight controller. After calibrating do the receiver test by selecting Receiver Test in the menu using S2 and S4 buttons. Next do the PI settings by setting the values as given in* Table 1 *:*

**Table 1:** Instructions for PI Settings:

Parameter	Roll (Aileron)	Pitch (Elevator)	Yaw (Rudder)
*P Gain*	*75*	*75*	*75*
*P Limit*	*50*	*50*	*20*
*I Gain*	*40*	*40*	*30*
*I Limit*	*20*	*20*	*10*

Once the PI settings are done then go to the Mode Settings, set the self-level to always. Then navigate to the miscellaneous settings by setting the Alarm 1/10 V to 108. (A 3-cell battery of 11.1 V has a value of 3.6 V per cell to denote the empty battery. Then set this value (in 1/10′s) to (3.6x3*10) = 108 when the supply voltage drops to 10.8 V the alarm will sound).

### ESC calibration

*Turn on the Flysky transmitter with the throttle to minimum and all the switches off, then move the throttle to maximum. Keep S1 and S4 buttons pressed together and then connect the battery, two beep sounds will be heard and then make the throttle to minimum, and a single beep sound will be heard. This will complete the ESC calibration. Now ARM the quadcopter by moving the throttle stick to the bottom left corner. Now by increasing the throttle the motors will start rotating. Make sure you don't attach the propellers. Check the motor rotation and make sure it is according to the X configuration. If the rotation is wrong, then interchange the last two wires of the ESC connected to the motor.*

#### Raspberry pi

For setting up the raspberry pi, connect the raspberry pi to a display.

*Start by writing a SD card with the raspberry pi operating system using the raspberry pi imager tool. Then insert the SD card in the SD card slot of Raspberry Pi and power up the raspberry pi. Follow the on-screen instructions and set up the raspberry pi. After setting up the raspberry pi, open the terminal and install all the following libraries: tkinter, DHT11, PIL, OpenCV, panda, sklearn, keras, pynmea and smtplib.*

*For using the internet using the GSM module, first disable the serial option from the interfaces tab, and we connect the raspberry pi to the internet with the help of wifi or ethernet, install PPP software using the sudo command in the terminal. After installation create a PPP PEER Configuration file. For creating the new peer file, login as a root user in the terminal and open the peers folder and make a new text file in the name of rnet. Copy the rnet text files included in the design file summary in the one which is created on the raspberry pi. In this file change the APN of the service provider. Also set the communication port accordingly by checking on which port it is connected. In the terminal by running the following command sudo pon rnet will turn on the internet on the raspberry pi. We can test this by opening the browser and searching on internet or else use of a Wi-Fi dongle and attaching it to drone can be also done.*

*As the raspberry pi has one Rx and Tx pins, and two of these pins requires so using a software based serial port module GPIO pins will behave as Rx and Tx. For that we must first install soft_uart and using the default pins i.e., GPIO pin 17 as Tx and GPIO pin 27 as Rx.*

*Test the functionality of each sensor/module before running the final file. Install the VNC Server application on raspberry pi and VNC viewer on the master computer. Run ipconfig command in the terminal of the raspberry pi and note down the IP address of the raspberry pi. Insert this IP address in VNC viewer to take the control of raspberry pi remotely. This step needs to be done once only if the IP is not changed of the raspberry pi. Whenever the system is turned on we can connect it directly with the help of VNC viewer. After testing individual sensors, run the forest-copter micro python file and see the GUI pop up showing all the required information such as temperature, humidity, camera feed, gps coordinates and prediction of forest fire. For making the program run after the startup we need to edit the.bashrc file on the raspberrypi. For editing the.bashrc file run the following command for opening the.bashrc file “sudo nano /home/pi/.bashrc”. We go to the last and add the following line to it. (“sudo python /home/pi/forestcopter/* *forest-cop-ter.py* *”). After every startup this file will be executed.*

#### Controlling drone through GUI

**Fig. 8:**
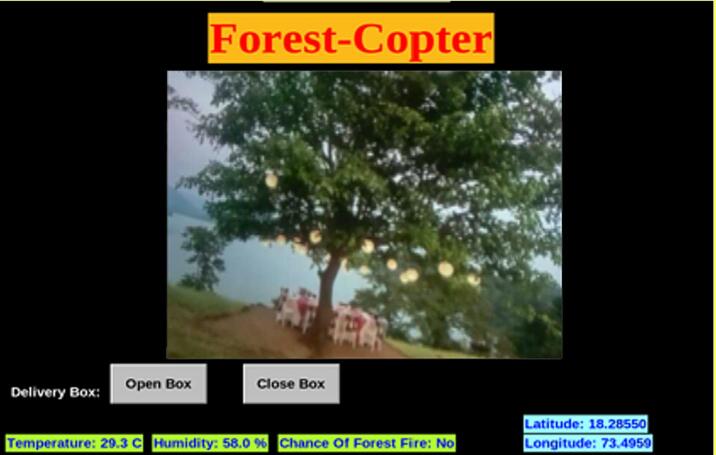
Graphical User Interface.

*To start the drone, the drone operator would first have to turn on his system and through VNC viewer connect with the drone which would start the GUI as explained in the Build Instructions. The GUI's aim is to give the operator a smooth experience in handling and observing the different characteristics designed on the drone. At the top of the GUI the name of the hardware is displayed in bold letters. Below it is a box that displays the surveillance streamed by the Raspberry Pi Camera in real time. Next, there are two buttons just below the surveillance to the left for opening and closing of the first aid kit attached to the drone. Followed by this, the data from the sensors along with the forest fire prediction is dispayed. The temperature and humidity data from DHT 11 sensor, and the prediction based on this real-time data whether there is a chance of a forest fire is displayed on the bottom left corner. Lastly, the coordinates of the drone displayed on the bottom right corner of the GUI received from the Neo-6 M GPS Module. Below* Fig. 8 *is the GUI developed working in real time.*

## Validation and characterization

### AI (Software)

*An AI model was developed to predict forest fires using meteorological data, specifically humidity and temperature. The dataset used for training and evaluation was sourced from the UCI repository* [23] *. To overcome challenges in dataset size and availability, synthetic data was generated using GANs. The model's performance was evaluated on both the original and augmented datasets. Additionally, the model was implemented on a drone platform, where real-time data from a DHT11 sensor was collected and processed for predictions, continuously updating the model with the acquired real-time data.*

#### Dataset

*Predicting forest fires based solely on humidity and temperature is challenging due to the numerous contributing factors like windspeed, soil moisture, and fuel presence. To address data scarcity, synthetic data was generated using Generative Adversarial Networks (GANs) to create a diverse dataset with multiple factors. The synthetic data included variables such as FFMC, DMC, DC, ISI, temperature, humidity, wind speed, rain index, and area burnt. By augmenting the dataset from 500 to 5000 samples, the GAN model improved accuracy. The GAN model consists of a generator network that generates new data samples and a discriminator network that distinguishes real from fake data. This approach enhances forest fire prediction using deep learning and real-time data collected by sensors.*

#### Architecture for GANs model

*The GAN model used consists of two main components: a generator network and a discriminator network. The generator network is responsible for generating new data samples, while the discriminator network is used to distinguish between real and fake data.*

#### The equations for Generative Adversarial network (GAN)

The generator, G, is a neural network that takes in a random noise vector, z, and generates a sample, G(z). The goal of the generator is to produce samples that are indistinguishable from real data.

*The discriminator, D, is a neural network that takes in a sample, either from the real data or from the generator, and outputs a scalar representing the probability that the sample is real. The goal of the discriminator is to correctly identify whether a sample is real or fake.*

*The generator and discriminator are trained in an adversarial manner, with the generator trying to produce samples that can fool the discriminator and the discriminator trying to correctly identify the fake samples. The training process can be described by the following loss functions:*

The generator's loss function, L_G, is typically the negative of the discriminator's output for fake samples, -log(1-D(G(z))). The generator wants to maximize this loss, which corresponds to fooling the discriminator.

*The discriminator's loss function, L_D, is typically the binary cross-entropy loss between the discriminator's output for real samples and a label of 1, and the discriminator's output for fake samples and a label of 0. The discriminator wants to minimize this loss, which corresponds to correctly identifying real and fake samples.*

The combined training process can be described by the following equations:

G is trained to minimize L_G = -log(D(G(z))).

D is trained to minimize L_D = -(log(D(x)) + log(1-D(G(z)))).

The generator and discriminator are trained alternately, with the generator's parameters being held *fixed* during the training of the discriminator and vice-versa.

#### Ann

*An Artificial Neural Network (ANN) is trained on the collected data to predict forest fires based on temperature and humidity relative to the burnt area. ANNs are chosen for their ability to handle complex, nonlinear relationships in tabular data. By adjusting weights and biases during training, ANNs can effectively model such relationships. Establishing a threshold to determine forest fire occurrence based on humidity and temperature relative to the burnt area was a challenging task, addressed through data analysis and visualization methods.*

The equations for Artificial Neural Network Model.

The mathematical equation for this model can be *represented* as:

y = relu(W1x + b1)
y = relu(W2y + b2)
y = relu(W3y + b3)
y = relu(W4y + b4)

where,

• ‘x’ is the input
• W1, W2, W3 and W4 are the weight matrices
• b1, b2, b3, b4 are the bias terms
• The relu function is defined as relu(x) = max(0,x)

#### Results and observations

##### GANs

**Fig. 9:**
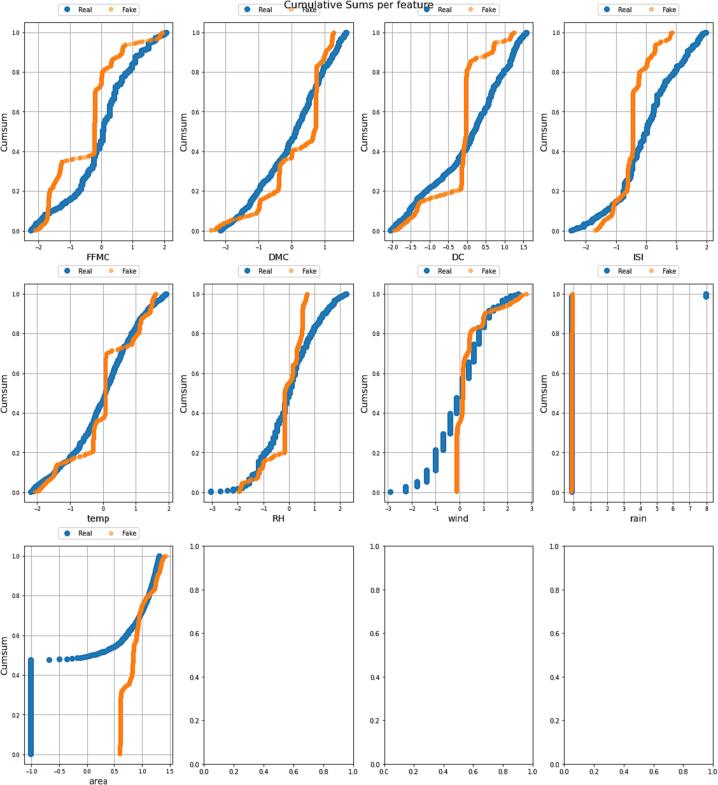
Cumulative Sums per feature graph of Real and Synthetic Data.

**Fig. 10:**
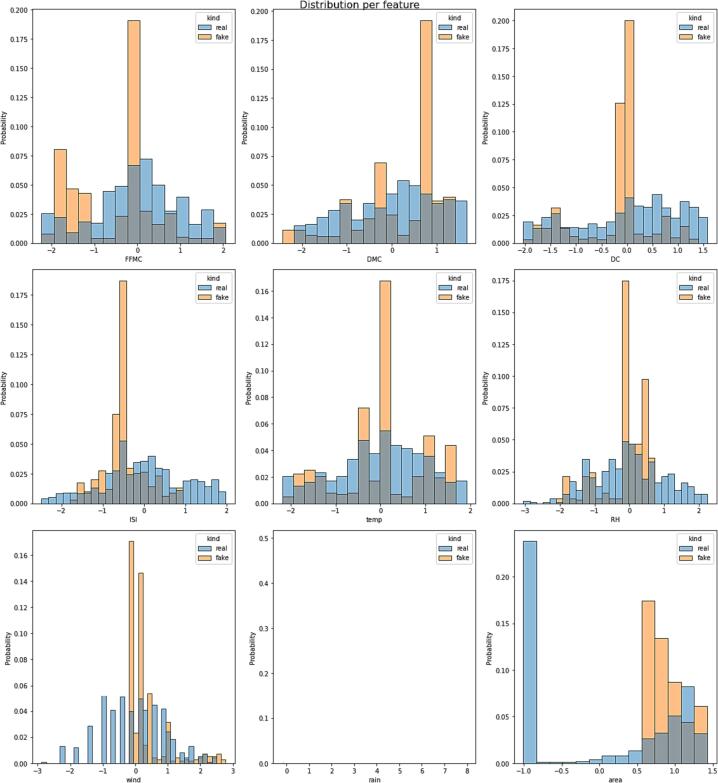
Distribution per feature graph of Real and Synthetic Data.

**Fig. 11:**
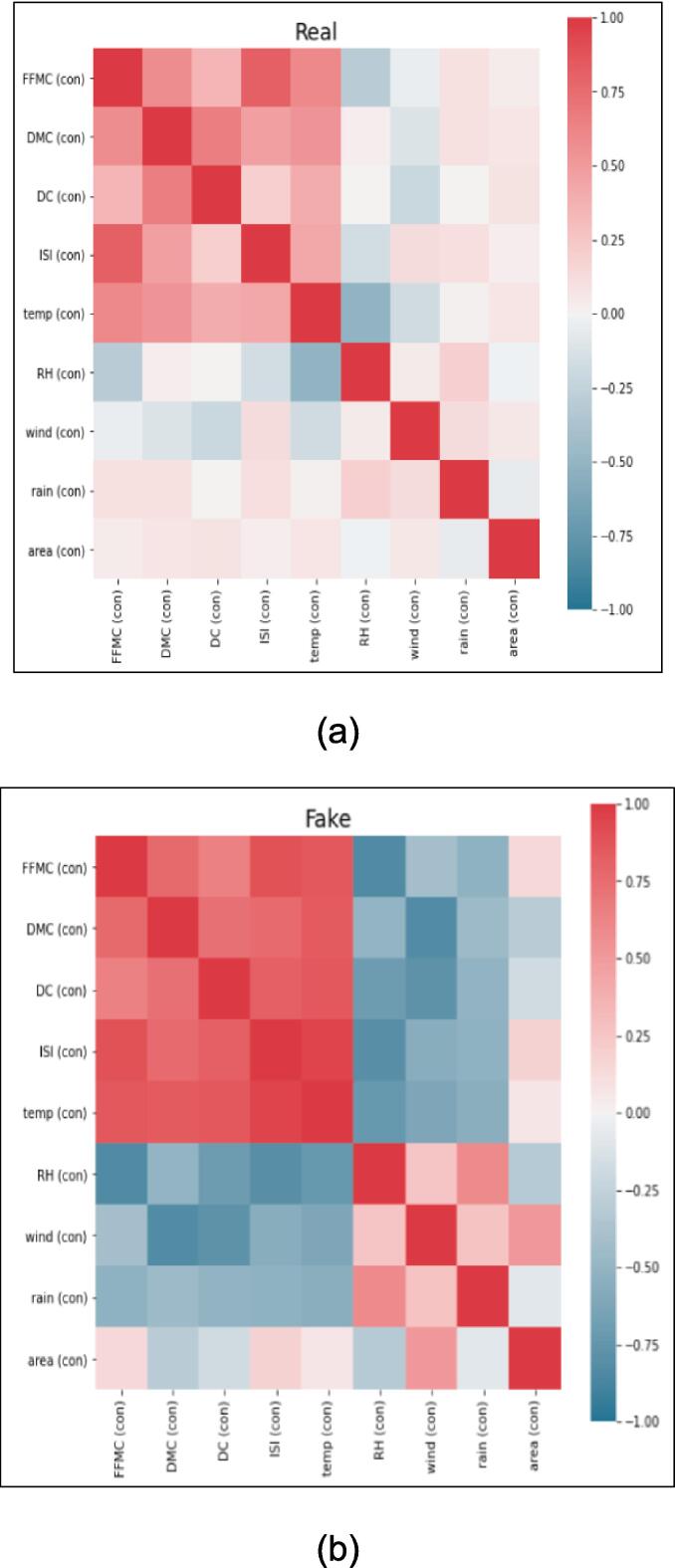

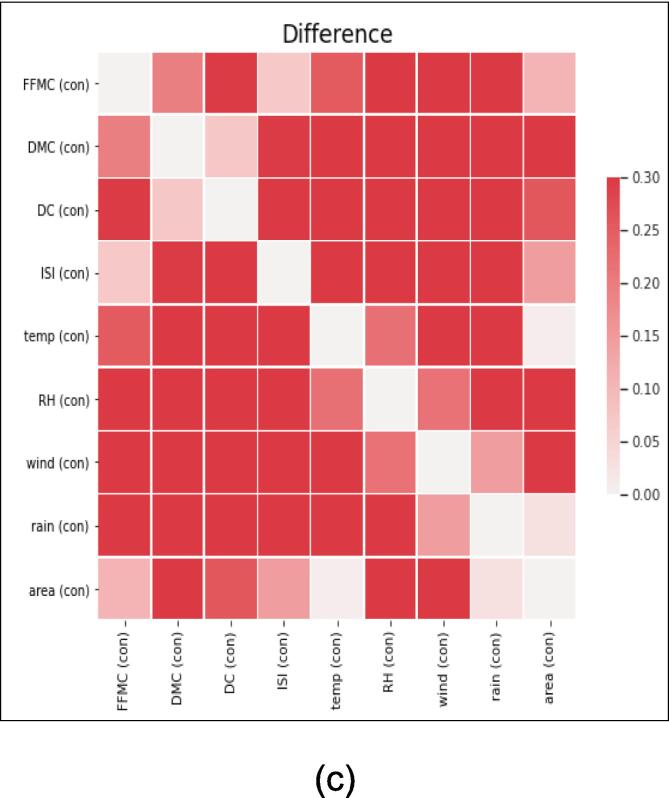
Heat map of (a) Real (b) Synthetic and (c) Difference between Real and Fake Data.

**Fig. 12:**
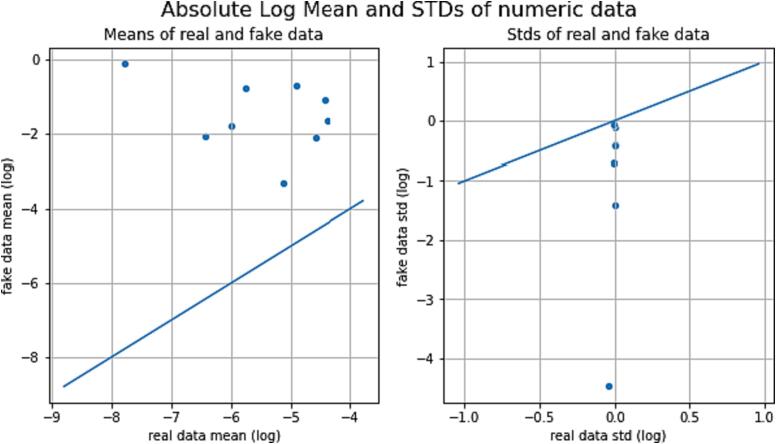
Absolute Log Mean and Standard Deviation of numeric data.

*In this section, we present a comprehensive analysis and comparison of real and synthetic data to explore their trends and variability. The evaluation focuses on key parameters such as temperature, relative humidity, area, and rain, which serve as essential input features for the deep learning model. The visual representations in* Fig. 9, Fig. 10, Fig. 11, Fig. 12 *shed light on the characteristics of the data and offer valuable insights into the performance of the generated synthetic data in comparison to actual data points.*

*Below* Fig. 9 *depicts the cumulative sum per feature graph, presenting the progression of features over time. The graph illustrates the sum of features between fake and real data, allowing us to understand their values at different points. The distinct patterns exhibited by the fake data indicate similarities with the real data, albeit with certain diversities that enhance the deep learning model's predictive capabilities in real-time. Notably, the data of temperature, relative humidity, and area exhibit intriguing characteristics, with humidity and temperature matching the trend of real data, while negative values in the area are eliminated due to their impracticality.*

*Below* Fig. 10 *provides insight into the likelihood prediction of data points based on real data and its comparison across a range of features. The differences in probability between real and fake data at specific points highlight potential outliers that warrant further investigation and elimination. Additionally, the absence of a distribution graph for the 'rain' parameter is justified by its constant cumulative sum, suggesting a homogeneous dataset with minimal variability across the data points.*

*The heatmap presented in* Fig. 11 *below showcases the correlation between real and synthetic data, accentuating the differences between the two datasets. However,* Fig. 9 *(c) indicates a negligible disparity, supporting the authenticity and validity of the generated synthetic data.*

*Below* Fig. 12 *illustrates how each feature performed concerning fake data points generated in comparison to actual data points. Utilizing standard deviation, the graph represents the data variation from the mean value. The positive correlation between the mean values of real and fake data suggests a close resemblance, with slightly higher means observed in the fake data. Furthermore, consistently lower standard deviation values in the fake data indicate minor differences in mean and variability compared to the real data.*

##### ANN

**Fig. 13:**
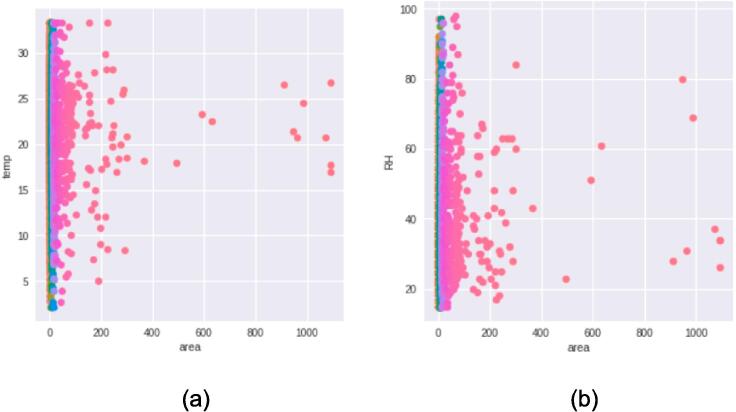
Scatter plot of Area of Forest Fire Burnt with respect to (a) Temperature (b) Humidity.

*In this section, we present a comprehensive evaluation of the performance metrics of our Artificial Neural Network (ANN) model for forest fire prediction. To optimize the model's sensitivity to forest fire detection, a scatter plot shown below in* Fig. 13 *is employed to determine an appropriate threshold, enabling timely alarm activation when required. Additionally,* Table 2 *provides essential validation results, demonstrating the significant performance enhancement achieved by incorporating synthetic data in the model training process.*

**Table 2:** Model Performance on Original and Synthetic Dataset.

Sr.No.	Metric	Synthetic Dataset	Original Dataset
1.	Test Accuracy	0.907	0.885

Following are capabilities and limitations of the AI model (ANN and GANs):

Capabilities:

• Provides a solution to limited available data.
• Can be trained on a variety of datasets.
• Can make predictions in real-time.
• Can improve over time as it is trained on more data.

Limitations:

• Requires large amounts of data to train effectively.
• Can struggle with small or noisy datasets.
• Can be prone to overfitting if not properly regularized.
• The accuracy of the model is dependent on the quality of input data and the complexity of the model.
• Can be sensitive to the choice of hyperparameters.
• Is limited to only a particular forest region.

### Hardware

#### Hardware validation

*This section delves into our drone's performance verification, including weight load capacity, flight stability, and flight duration. Through rigorous testing and calculations, we ensure optimal operational efficiency and stability, while also exploring the intricate interplay of battery capacity and flight time. Additionally, the limitations and capabilities of the hardware has been listed out in* Table 3 *.*

**Table 3:** Capabilites and Limitations of the components used while designing the Quadcopter.

Sr.No.	Component	Capabilities and Limitations
*1.*	*F450 Quadcopter*	*The system has a flight autonomy of 90 min with a 5200mAh battery, along with the frame carrying capacity up to 2.5 kg at a* max *speed of 20 m/s, but reduced flight accuracy at maximum weight.*
*2.*	*Flysky 6CH Controller*	*The system offers 6 channels for flight control customization, a range of around 1.5 km and long-lasting operation with a large battery capacity; however, its accuracy can be affected by signal quality, interference, and operator skill, the binding process may be challenging, and it requires more batteries due to higher power consumption.*
*3.*	*KK2.1 Multi-rotor LCD Flight Control Board*	*The system can control the drone's flight with an automatic stabilization function to prevent drifting and support for various flight modes, but it lacks an inbuilt GPS, rendering it unable to navigate and hover.*
*4.*	*30A ESC*	*The system can control the speed of the brushless motors with a maximum current of 30A and an operating voltage of 2-3S LiPo battery, but calibrating the ESCs might pose difficulties for newcomers.*
*5.*	*0845 Propeller 8x4.5 For Drone Kit (AntiClockwise, Clockwise)*	*The system's 8x4.5-inch propellers provide stability and lift, allowing the drone to withstand high speeds and winds, but it is also susceptible to wind interference and produces more noise during operation.*
*6.*	*A2212 1000KV Brushless Motor*	*The system can provide a maximum thrust of around 800gm and reach a maximum speed of approximately 27,000 RPM; nevertheless, there is a risk of motor overheating and failure due to high current and power draw.*
*7.*	*5200 mAh LiPo Battery*	*The system offers a flight time of around 90 min and a recharge time of approximately 2 h; nevertheless, its performance may be impacted by the battery's load capacity.*
*8.*	*Raspberry Pi 4*	*The system includes a 1.5 GHz 64-bit quad-core processor, onboard Wi-Fi, Bluetooth 5.0, Gigabit Ethernet, and support for various sensors, but it may experience data transfer issues due to power supply fluctuations, performance limitations with memory capacity, and heating problems during heavy use.*
*9.*	*UBLOX NEO-6 M GPS module*	*The system has a high-sensitivity receiver with an accuracy of around 2.5 m, capable of tracking up to 22 satellites, but its performance may be affected in certain environments with lower signal strength.*
*10.*	*SIMCOM GSM SIM900A Module*	*The system features a quad-band GSM/GPRS module enabling call making and receiving as well as SMS messaging capabilities, but it has a limited data transfer rate and higher power consumption during data transmission.*
*11.*	*Speaker*	*The system can provide clear and loud sound for communication with people in the forest, but it has limitations in sound quality and limited volume, which may affect its performance in noisy environments.*
*12.*	*Microphone*	*The system can capture clear and accurate audio for communication with people in the forest, but it is limited by the microphone's directionality and has a restricted frequency response.*
*13.*	*Raspberry Pi Camera*	*The system includes an 8-megapixel camera for high-resolution surveillance applications in the forest, but its performance can be affected by feed quality, limited frame rate, and low light conditions.*
*14.*	*Adafruit DHT11 sensor*	*The system can measure temperature and humidity with an accuracy of around 2% for humidity and 0.5 °C for temperature, making it suitable for forest data collection, but it is limited by slow response time and a restricted operating range for the sensor.*
*15.*	*TowerPro Micro Servo SG90*	*The system can rotate up to 180 degrees with a torque of around 9 kg/cm, facilitating the opening of the first aid kit lid, but its accuracy can be influenced by temperature and other factors, and the motor may stall or get damaged under heavier loads.*

##### Weight load carrying capacity

*We conducted a thorough validation of our drone's weight carrying capacity to ensure safe and stable flight performance. With a specified payload weight of 1600 g, each of the four motors produces a robust thrust of 800 g. Applying the industry-standard guideline of a 2:1 thrust-to-weight ratio for stable flight* [24] *, we calculated a total thrust of 3200 g. Comparing this to the payload weight, we achieved an impressive thrust-to-weight ratio of 2:1, surpassing the required threshold. This meticulous validation process ensures that our drone is equipped to handle the specified weight while maintaining stability and controlled maneuverability during its operational missions.*

##### Flight stability

*As described in Section 6.1.1 the flight control calibration sequence employs variable-based adjustments to validate and enhance the drone's stability. By configuring button assignments (S1-S4) for navigation and menu access, and selecting the Quadcopter X mode (M) for motor layout, balanced thrust distribution is achieved. Motor rotation direction adherence (Rot) in accordance with the X configuration ensures optimal lift symmetry. Accelerometer calibration (ACC) on a level surface guarantees accurate attitude determination. Receiver testing (Rx) assesses signal reception and control fidelity. Precise proportional (P) and integral (I) gain settings from* Table 1 *optimize stability by fine-tuning control response. Activation of the self-level mode (SL) introduces automatic leveling for stable flight. Miscellaneous adjustments (Misc) like alarm voltage threshold contribute to power management. Employing these variable-calibrated parameters assures heightened stability, responsiveness, and controlled flight.*

##### Flight time

*In this section, we validate the flight time and performance of the drone under varying load conditions, considering factors such as battery capacity, discharge efficiency, and practical flight observations. The calculations and observations provide insights into the drone's running time and its responsiveness to different scenarios.*

• Battery Capacity: 5200mAh (5.2Ah)
• Discharge Efficiency: 85% (0.85)
• Battery Voltage: 12.6 V

Calculation of Flight Time:

• Average Current Draw (Ampere): TFW × Power-to-Weight Ratio / Battery Voltage [25]
• Flight Time (hours): Capacity × Discharge Efficiency / Average Current Draw [25]

Drone Performance - No Load:

• Total Flight Weight (TFW): 0.5 kg
• Average Current Draw (ACD): 3.17A
• Flight Time: 1.39 h (84 min)

Drone Performance - With Load:

• Total Flight Weight (TFW): 1.5 kg
• Average Current Draw (ACD): 9.5A
• Flight Time: 0.46 h (28 min)

Additional Observations:

• Flight Time with Load: Theorotical load test yielded around 28 min, but real-world testing indicated approximately 45 min in open space.
• No-load Flight Time: Theoretical calculation suggested 84 min, while actual open-space testing demonstrated about 90 min.
• Influence of Small Propellers: The use of smaller propellers resulted in lower current consumption and extended flight times.
• Weather Conditions Impact: Normal weather conditions contributed to the achieved flight durations.

*Please note that these are approximate figures based on the experiments done and the actual performance may vary depending on various factors such as weather conditions, flight conditions, and maintenance of the hardware.*

## Ethics statements

The work did not involve any human or animal subjects, nor data from social media platforms.

## Declaration of Competing Interest

The authors declare that they have no known competing financial interests or personal relationships that could have appeared to influence the work reported in this paper.

## References

[1] Disaster Philanthropy. (2022). 2022 International Wildfires. Available at: https://disasterphilanthropy.org (Accessed on: Feb 4, 2023).

[2] *National Centers for Environmental Information. (2022). U.S. monthly report: Fire. Available at: https://www.ncei.noaa.gov (Accessed on: Feb 4, 2023)*.

[3] *DowntoEarth.org.in. (n.d.). India's forest fires are getting bigger and hotter, like the rest of the world. Available at: https://www.downtoearth.org.in. (Accessed on: Feb 4, 2023)*.

[4] *Wired. (n.d.). Why humans totally freak out when they get lost. Available at: https://www.wired.com (Accessed on: Feb 4, 2023)*.

[5] Jankovský M., Allman M., Allmanová Z. (2019). What Are the Occupational Risks in Forestry? Results of a Long-Term Study in Slovakia. Int. J. Environ. Res. Public Health.

[6] *Encyclopedia (n.d.). Available at: https://encyclopedia.pub (Accessed on: Feb 4, 2023)*.

[7] *van Hensbergen, H., & Cedergren, J. (2020). Forest-related Disasters—Three Case Studies and Lessons for Management of Extreme Events. Forestry Working Paper No. 17. Rome: FAO. Available at: https://www.fao.org (Accessed on: Feb 4, 2023)*.

[8] Blennow K., Persson E. (2013). Living with Storm Damage to Forests.

[9] *ArduPilot. Available at: https://ardupilot.org/ (Accessed on: Feb 4, 2023)*.

[10] *PX4. Available at: https://px4.io/ (Accessed on: Feb 4, 2023)*.

[11] *DJI Matrice 600 Pro. Available at: https://www.dji.com (Accessed on: Feb 4, 2023)*.

[12] *Autel Robotics EVO II Collections. Available at: https://shop.autelrobotics.com (Accessed on: Feb 4, 2023)*.

[13] *A. User, OV7670 Arduino Camera Sensor Module Framecapture Tutorial. Available at: https://www.instructables.com, (Accessed on: Feb 4, 2023)*.

[14] *Beagleboard Community, BeagleBone HD Camera Cape. Available at: https://elinux.org, (accessed date)*.

[15] *GoPro, HERO 10 Black - Bones. Available at: https://gopro.com, (Accessed on: Feb 4, 2023)*.

[16] Wikipedia contributors, “LoRa,” Wikipedia, The Free Encyclopedia, Available at: wiki/LoRa, (Accessed on: Feb 4, 2023).

[17] *u-blox. (n.d.). u-blox | NEO-M8 Series. Available at: https://www.u-blox.com (Accessed on: Feb 4, 2023)*.

[18] *Adafruit Industries, LLC. (n.d.). Adafruit Ultimate GPS - 66 channel w/10 Hz updates - Version 3 [ADA746]. Available at: https://www.adafruit.com (Accessed on: Feb 4, 2023)*.

[19] *Robu.in. (n.d.). DJI NAZA GPS Module. Available at: https://robu.in (Accessed on: Feb 4, 2023)*.

[20] *Simplefoc. (n.d.). BLDC Motors. Available at: https://docs.simplefoc.com. (Accessed on: Feb 4, 2023)*.

[21] *BME280. “Bosch BME280.” Mouser Electronics, Available at: https://www.mouser.in (Accessed on: Feb 4, 2023)*.

[22] *Si7006-13-20-21-34. “Device Si7021-A20-GM.” Silicon Labs, Available at: https://www.silabs.com (Accessed on: Feb 4, 2023).*.

[23] *[Cortez and Morais, 2007] P. Cortez and A. Morais. A Data Mining Approach to Predict Forest Fires using Meteorological Data. In J. Neves, M. F. Santos and J. Machado Eds., New Trends in Artificial Intelligence, Proceedings of the 13th EPIA 2007 - Portuguese Conference on Artificial Intelligence, December, Guimarães, Portugal, pp. 512-523, 2007. APPIA, ISBN-13 978-989-95618-0-9*.

[24] Selig M.S. (2014). Real-time flight simulation of highly maneuverable unmanned aerial vehicles. J. Aircr..

[25] *Fly That Drone. Flight Time Calculator. Available at: https://flythatdrone.com/tools/flight-time-calculator/ (Accessed on: [Aug 3, 2023])*.

[26] Liardon J.-L., Hostettler L., Zulliger L., Kangur K., Gujja Shaik N.S., Barry D.A. (2018). Lake imaging and monitoring aerial drone. HardwareX.

[27] Ryu J.H. (2022). UAS-based real-time water quality monitoring, sampling, and visualization platform (UASWQP). HardwareX.

